# Molecular detection of filarioid nematodes (Nematoda: Onchocercidae) in wild mammals from different Brazilian biomes

**DOI:** 10.1017/S0031182025101042

**Published:** 2025-11

**Authors:** Matheus de Souza Santana, Aline Pedroso Lorenz, Maite Cardoso Coelho da Silva, Gediendson Ribeiro de Araujo, Antonio Carlos Csermak-Junior, Thyara de Deco-Souza Araujo, Herbert Patric Kellermann Cleveland, Ivanise Paula Sobota, Leila Sabrina Ullmann, Guilherme Gomes Verocai, Carlos Alberto Nascimento Ramos

**Affiliations:** 1 Department of Epidemiology and Public Health, Federal Rural University of Rio de Janeiro, Seropédica, RJ, Brazil; 2 Faculdade de Medicina Veterinária e Zootecnia, Universidade Federal de Mato Grosso do Sul, Campo Grande, MS, Brazil; 3 Instituto de Biociências, Universidade Federal de Mato Grosso do Sul, Campo Grande, MS, Brazil; 4 Biotério Central, Universidade Federal de Mato Grosso do Sul, Campo Grande, MS, Brazil; 5 Reprocon Institute, Campo Grande, MS, Brazil; 6 Department of Veterinary Pathobiology, College of Veterinary Medicine and Biomedical Sciences, Texas A&M University, College Station, TX, USA

**Keywords:** biodiversity, *Brugia*, *Dirofilaria*, Filarioidea, South America, Zoonosis

## Abstract

Parasitic nematodes within Onchocercidae are a diverse group transmitted by hematophagous arthropods. This study investigated the molecular occurrence of filarioid nematodes in 93 wild mammals from the Amazon, Cerrado and Pantanal biomes in Brazil, based on the analysis of the mitochondrial genes 12S ribosomal DNA gene (12S rDNA) and cytochrome c oxidase subunit I gene (COI). Conventional polymerase chain reaction (cPCR) targeting the 12S rDNA gene yielded positive results in 14·44% (13/93) of the samples, including 9·86% of jaguars (7/71), 50% of pumas (1/2), 12·5% of giant anteaters (1/8), 50% of ocelots (1/2) and 60% of crab-eating foxes (3/5). Among the 12S-positive samples, 46% (6/13) also tested positive for the COI gene; however, only 1 sequence was suitable for further analysis. Phylogenetic analyses based on 12S gene sequences revealed 4 distinct lineages within the family Onchocercidae. Groups Ia and Ib, composed of Cerrado and Pantanal sequences from jaguars, formed sister clades to *Brugia pahangi* and *Malayfilaria sofiani*, respectively. The sequence from the giant anteater (Group Ic) was more divergent, forming a sister clade to species of the genera *Malayfilaria, Wuchereria,* and *Brugia*. Group II included sequences closely related to *Dirofilaria immitis* and *D. striata*, encompassing samples from crab-eating foxes, ocelots and a puma. These findings suggest that several wild mammal species may serve as reservoirs for previously uncharacterized Onchocercidae nematodes. Our findings expand the existing knowledge on host associations of filarioid nematodes infecting wild mammals from the Pantanal, the Cerrado and the Amazon Rainforest.

## Graphical abstract

**Figure:**
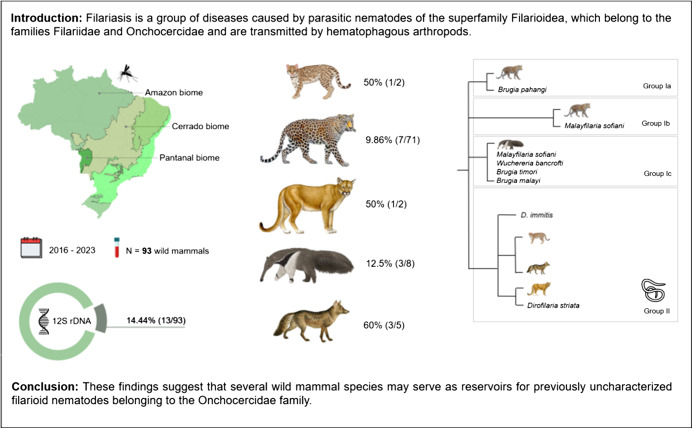

## Introduction

Filariasis is defined as a group of diseases caused by parasitic nematodes belonging to the superfamily Filarioidea, which is classified into the families Filariidae and Onchocercidae, which are considered a relatively small group within the extensive phylum Nematoda (Anderson, 2000). Filarioid nematodes are transmitted by mosquitoes (Culicidae) as well as by black flies (Simuliidae) and other blood-feeding arthropods (Ludlam et al. 1970; Anderson, 2000; McCall et al. 2008; Lefoulon et al. 2017). In their vertebrate definitive hosts, these parasites produce microfilariae that can be found in the bloodstream, cutaneous tissues, or mucous membranes, becoming available for the infection of new competent arthropod vectors (Anderson, 2000; Otranto et al. 2009, 2013). Various genera and species of filarioid nematodes are known to have relevant medical and veterinary importance (Morales-Hojas et al. 2001). In humans, they are the primary etiological agents of lymphatic filariasis and river blindness, among other diseases (Anderson, 2000).

The genus *Dirofilaria* includes more than 20 species associated with mammalian definitive hosts, and most species use mosquitoes as intermediate hosts (Anderson, 2000). However, the most relevant species in veterinary medicine are *Dirofilaria immitis* and *Dirofilaria repens*. The former is responsible for causing a cardiopulmonary disease, commonly known as heartworm disease, which may lead to congestive right heart failure, potentially resulting in death. In contrast, in other parts of the world, such as the Middle East and particularly in Eastern European countries, Dirofilaria repens is the most endemic parasitic nematode (McCall et al. 2008; Capelli et al. 2018; Genchi and Kramer, 2020). This species causes subcutaneous infections characterized by the presence of nodules (Albanese et al. 2013). Although less pathogenic, these infections seem to have a higher zoonotic potential compared to those caused by *D. immitis* (Genchi and Kramer, 2017). In Brazil, several species within the genus *Dirofilaria* have been reported, including *D. immitis* (Dantas-Torres et al. 2020; Barbosa et al. 2023; Zanfagnini et al. 2024), often associated with domestic and wild carnivores; *Dirofilaria incrassata* (Vicente et al. 1997), *D. repens* (Noronha et al. 2002; Moraes et al. 2017), *Dirofilaria spectans* (Vicente et al. 1997; Noronha et al. 2002) and *Dirofilaria striata* (Lentz and Freitas, 1937; Vicente et al. 1997).

The genus *Brugia* includes around 10 species associated with mammal definitive hosts and mosquito vectors (Xie et al. 1994). Out of these, *Brugia malayi* and *B. timori* are the most significant to public health, serving as the primary agents of lymphatic filariasis in South and Southeast Asia, as well as historically in Oceania. Infections by these parasites can lead to various clinical manifestations, particularly affecting the lymphatic system, such as lymphedema, hydrocele, fever, chills, lymphadenitis, skin ulcers, pain and tenderness (Anderson, 2000; Tan et al. 2011; Gordon et al. 2018). Although cases of *Brugia*-induced lymphatic filariasis have been reported in North America, there is evidence suggesting underreporting (Elenitoba-Johnson et al. 1996). In contrast, in Brazil, the disease in humans is primarily caused by *Wuchereria bancrofti*, phylogenetically closely related to *Brugia* (GOV.BR – https://www.gov.br/saude/pt-br/assuntos/saude-de-a-a-z/f/elefantiase). In the Americas, the following species from the genus *Brugia* have been reported: *B. beaveri, B. guyanensis, B. lepori* and other not fully characterized species (Ash and Little 1964; Orihel, 1964; Schlesinger et al. 1977; Eberhard 1984; Beaver and Wong, 1988; Eberhard et al. 1991; Moraes et al. 2017; Kulpa et al. 2023).

Understanding the health risks faced by wild animals is essential for preventing the emergence and spread of parasitic diseases that may threaten their conservation. Furthermore, other factors associated with anthropization, such as poaching, vehicle collisions on highways and habitat loss or destruction due to the expansion of agricultural and livestock activities, also pose significant threats (Sanderson et al. 2002; Rodden et al. 2004; Michalski and Peres, 2005; Paula et al. 2008). This is particularly relevant in the neotropical region, where significant gaps in knowledge exist regarding the diversity of onchocercid species due to the wide range of available hosts and the diversity of biomes. Therefore, the present study aimed to investigate the molecular occurrence of filarioid nematodes in wild mammals of the Amazon Rainforest, Cerrado and Pantanal biomes in Brazil.

## Materials and methods

### Sampled species and study area

**Figure 1.**
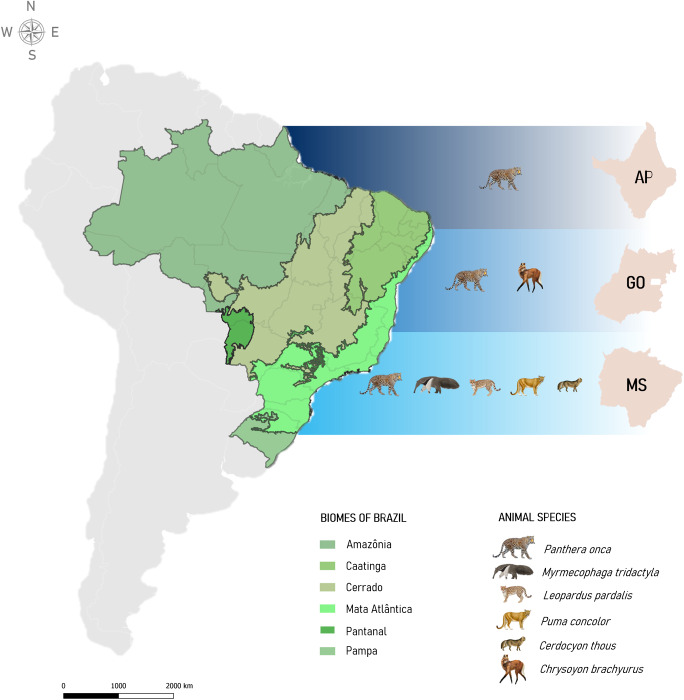
Distribution of sampled wild mammals captured by the state of Brazil (AP, Amapá, GO, Goiás and MS, Mato Grosso do Sul). The map also depicts the different biomes from where samples were collected.

Six wildlife species were sampled from 2016 to 2023, overall, including 71 jaguars (*Panthera onca;* Carnivora; Felidae), 2 pumas (*Puma concolor* Carnivora; Felidae), 8 giant anteaters (*Myrmecophaga tridactyla*; Xenarthra; Myrmecophagidae), 5 maned wolves (*Chrysocyon brachyurus*; Carnivora; Canidae), 5 crab-eating foxes (*Cerdocyon thous*; Carnivora; Canidae) and 2 ocelots (*Leopardus pardalis*; Carnivora; Felidae). Whole blood with EDTA was collected from a total of 93 animals, of which 57 were free-living and 36 were kept in captivity. Animal capture was conducted with the assistance of a licensed and registered veterinarian technical team with the Regional Council of Veterinary Medicine and Animal Health. Jaguars and pumas were captured using foot-snare traps (Araujo et al. 2021), and the giant anteaters were captured by active search, followed by restraint with a catcher pole. Animals were sedated using the association of medetomidine (0·08–0·1 mg kg^−1^; IM) and ketamine (5 mg kg^−1^; IM) (Araujo et al. 2021; Araújo et al. 2023). The samples were collected in the municipalities of Rio Negro (19°44’19.7”S 54°98’11.8”W), Bodoquena (20°54’09.1”S 56°71’34.2”W), Aquidauana (20°45’18.2’’S 55°78’21.0’’W), Corumbá (19°00’80.3’’S 57°64’35.5’’W), Miranda (19°52’40.3’’S 57°02’90.1’’W), in the state of Mato Grosso do Sul (Cerrado and Pantanal biomes), as well as in the states of Amapá, on Maracá-Jipioca Island (2°04’92.3’’S 50°45’06.6’’W) (Amazon biome), and Goiás at the Onça-Pintada Institute (17°89’19.3’’S 53°00’81.4’’W) and in Emas National Park (17°92’18.4’’S 53°00’48.7’’W) (Cerrado biome) (Figure 1). Whole blood samples were stored at –80 °C until further processing.

### Molecular analyses

Genomic DNA was extracted from whole blood samples following a previously published protocol (Araujo et al. 2009). Subsequently, conventional PCR (cPCR) was performed on the extracted DNA samples to amplify the mammalian endogenous gene *GAPDH* (glyceraldehyde-3-phosphate dehydrogenase) (Kojima et al. 2004) to verify the presence of amplifiable DNA. Samples that tested positive in this cPCR assay for mammalian endogenous genes were subjected to screening by cPCR assays for the detection of filarioid nematodes, through amplification of the 12S rDNA gene (Casiraghi et al. 2004) and characterization with the cytochrome c oxidase subunit 1 (COI) gene (Casiraghi et al. 2001).

All cPCR assays were performed using 2 μL of DNA (approximately 50 ng) in a mixture containing 1·5 U Taq DNA Polymerase (Ludwig, Alvorada, Rio Grande do Sul, Brazil), PCR buffer (10X PCR buffer – 10 mM Tris-HCl, pH 8·3, 50 mM KCl), 0·5 mM of deoxynucleotides (dATP, dTTP, dCTP and dGTP) (Cellco, São Carlos, São Paulo, Brazil), 1·5 mM of Magnesium Chloride (Ludwig, Alvorada, Rio Grande do Sul, Brazil), 0·5 μM of each primer (Invitrogen^®^, Carlsbad, California, USA), and ultrapure sterilized water (Ludwig, Alvorada, Rio Grande do Sul, Brazil) q.s.p. 25 μL. DNA samples from dogs infected with *Cercopitifilaria bainae*, from the study by Soares et al. (2020), were used as positive controls. Ultrapure sterilized water (Ludwig, Alvorada, Rio Grande do Sul, Brazil) was used as a negative control.

The amplified products were separated by agarose gel electrophoresis 2% agarose gel stained with GelRed^®^ Nucleic Acid Gel Stain (Biotium, San Francisco, California, USA), using an electric current of 100 V/150 mA for 50 min. Agarose gels were exposed to ultraviolet light (Easy doc 100, Bio Agency, São Paulo, São Paulo, Brazil).

### Purification of amplicons and sequencing

PCR products were purified using ExoSAP-IT PCR Product Cleanup Reagent (Applied Biosystems™) and sequenced using the BigDye™ Terminator v3.1 Cycle Sequencing Kit (Thermo Fisher Scientific™, Waltham, MA, USA) and ABI PRISM 3130 DNA Analyzer (Applied Biosystems™, Foster City, CA, USA) (Sanger et al. 1997), with the same primer pairs from cPCR.

The sequences obtained were subjected to quality screening using Geneious software (Kearse et al. 2012) to assess the quality of the electropherograms and obtain consensus sequences from the alignment of the direct and inverse sequences. Subsequently, the BLASTn analysis tool (Altschul et al. 1990) was used to analyse the nucleotide sequences by comparing them with sequences previously deposited in the GenBank database (Benson et al. 2017).

### Phylogenetic analyses

After an initial analysis with BLAST, a database was created containing all available sequences from GenBank for the sequenced regions and taxonomic groups (search conducted in October 2023). Exploratory alignments by genus were performed to exclude highly divergent sequences (likely identification errors) or incomplete sequences from subsequent analyses. Multiple sequence alignments for each locus were performed using MAFFT v.7 (Katoh and Standley, 2013), with the auto option, followed by manual inspection. The best nucleotide substitution model was selected using the Bayesian Information Criterion (BIC) in the jModelTest v.2.1.7 software (Darriba et al. 2012).

Phylogenetic trees were reconstructed using maximum likelihood analysis (ML) and Bayesian inference (BA). ML analyses were performed using RAxML (Stamatakis 2014), utilizing the GTRGAMMA model, with support values estimated from 1000 bootstrap pseudoreplicates. For Bayesian analysis (BA), we used the BEAST 2.6.7 package (Bouckaert et al. 2019), employing the HKY substitution model, the strict molecular clock and the Yule Model. Our analysis consisted of 2 independent Markov Monte Carlo Chains (MCMC) runs, each covering 20 000 000 generations and sampling every 10 000. JmodelTest, RAxML and Beast analyses were performed on CIPRES Science Gateway (Miller et al. 2010). The convergence was confirmed by inspecting the log probability in the Tracer 1.7 program (Rambaut et al. 2018). Estimated effective sampling size values exceeding 200 indicated that the convergence was achieved. The maximum clade credibility tree was estimated with TreeAnnotator, part of the BEAST package. As burn-in, 10% of the first trees obtained were discarded. The trees were visualized and edited using the FigTree v1.4.4 software (http://tree.bio.ed.ac.uk/software/figtree/). Branches with bootstrap support ≥75% in the ML and a posterior probability ≥0·95 in BA analysis were considered strongly supported.

Pairwise ML distances (substitutions/site) were estimated in PAUP 4.0b10 (Swofford, 2002), with base frequencies calculated in JMODELTEST2 program (Guindon and Gascuel 2003; Darriba et al. 2012).

## Results

### Occurrence of filarioid nematodes in free-living and captive wildlife

Out of the 93 samples analysed, 90 (96·77%) tested positive for the mammalian endogenous gene GAPDH via cPCR. In the screening assays targeting filarioid nematodes, based on cPCR for the 12S rDNA gene, 14·44% (13/93) yielded positive results. These included 9·86% of jaguars (7/71), 50% of puma (1/2), 12·5% of giant anteaters (1/8), 50% of ocelots (1/2) and 60% of crab-eating foxes (3/5). Among the samples that tested positive for the 12S rDNA gene, 46% (6/13), including 42·85% of the jaguars (3/7), 100% of the puma (1/1), 100% of the giant anteaters (1/1) and 33·33% of the crab-eating foxes (1/3), also tested positive for the COI gene (Table 1). It is important to note that the high percentages observed in certain species are due to the low sample sizes. Only free-ranging animals tested positive for filarioid nematodes.

**Table 1.** List of animals that tested positive in the conventional PCR assays for the 12S rDNA and COI genes, along with information regarding identification (ID), species, sex, age group, location and sequence

Species	Sex	Age group	Habitat	Location
*P. onca*	Female	Adult	Free-living	Bodoquena-MS
*P. onca*	Male	Cub	Free-living	Corumbá-MS
*M. tridactyla*	Male	Adult	Free-living	Rio Negro-MS
*C. thous*	Male	Adult	Free-living	Rio Negro-MS
*L. pardalis*	Female	Adult	Captive	Mineiros-GO
*P. onca*	Male	Adult	Free-living	Rio Negro-MS
*P. onca*	Male	Adult	Free-living	Rio Negro-MS
*M. tridactyla*	Male	Adult	Free-living	Rio Negro-MS
*M. tridactyla*	Male	Adult	Free-living	Rio Negro-MS
*P. concolor*	Male	Adult	Free-living	Rio Negro-MS
*P. onca*	Male	Adult	Free-living	Corumbá-MS
*P. onca*	Male	Adult	Free-living	Miranda-MS
*P. onca*	Male	Adult	Free-living	Rio Negro-MS

GO, Goiás; MA, Maranhão; MS, Mato Grosso do Sul.

### Exploratory sequence analyses

All 12S rDNA-amplified samples yielded high-quality sequences; however, we encountered difficulties in obtaining high-quality sequences for the COI region under the tested conditions. All sequences were deposited in the GenBank database and assigned the following accession numbers: OR852676-87, PQ699718 and PQ699178. In the results of the identity analyses conducted by BLASTn, the hit with the highest percentage of identity was considered for filarioid detection based on the 12S rDNA target gene (Table 2).

**Table 2.** Percentage of BLASTn-associated identity of sequences of the family Onchocercidae detected in free-ranging and wild-caught wild animals in Brazil

Species/ID	GenBank accession numbers	Target gene	Query length (pb)	Query-coverage (%)	E-value	Identity (%)	GenBank accession numbers
*P. onca*/#2	OR852684	12S rDNA	431	99	2 × 10^−71^	92·08	*Malayfilaria sofiani* (KX944562)
*P. onca*/#5	OR852676	12S rDNA	431	100	9 × 10^−108^	90·94	*Malayfilaria sofiani* (KX944562)
*M. tridactyla*/#9	OR852683	12S rDNA	13 744	92	3 × 10^−130^	95·44	*Onchocerca ochengi* (AY462914)
*C. thous*/#13	OR852678	12S rDNA	513	99	2 × 10^−130^	94·25	*Dirofilaria immitis* (KP898738)
*L. pardalis*/#16	OR852679	12S rDNA	687	99	9 × 10^−142^	94·61	*Dirofilaria immitis* (KF707479)
*P. onca*/#36	OR852677	12S rDNA	13 675	99	5 × 10^−132^	92·94	*Brugia pahangi* (AP017680)
*P. onca*/#39	PQ699718	12S rDNA	13 675	98	5 × 10^−132^	92·83	*Brugia pahangi* (AP017680)
*C. thous*/#57	OR852680	12S rDNA	513	97	2 × 10^−138^	94·24	*Dirofilaria immitis* (MF059093)
*C. thous*/#58	OR852681	12S rDNA	513	96	1 × 10 ^−139^	94·28	*Dirofilaria immitis* (KF707479)
*P. concolor*/#59	OR852682	12S rDNA	687	99	1 × 10^−132^	92·75	*Dirofilaria immitis* (KF707479)
*P. onca*/#61	OR852685	12S rDNA	995	100	1 × 10^−127^	91·72	*Brugia malayi* (XR006227884)
*P. onca*/#67	OR852686	12S rDNA	995	96	4 × 10^−114^	87·5	*Brugia malayi* (XR006227884)
*P. onca*/#81	OR852687	12S rDNA	13 658	100	1 × 10^−107^	85·53	*Brugia timori* (AP017686)
*P. concolor*/#59	PQ699178	COI	13 675	99	0	92·15	*Dirofilaria repens* (NC029975)

### Phylogenetic analyses of 12S rDNA and COI genes

**Figure 2.**
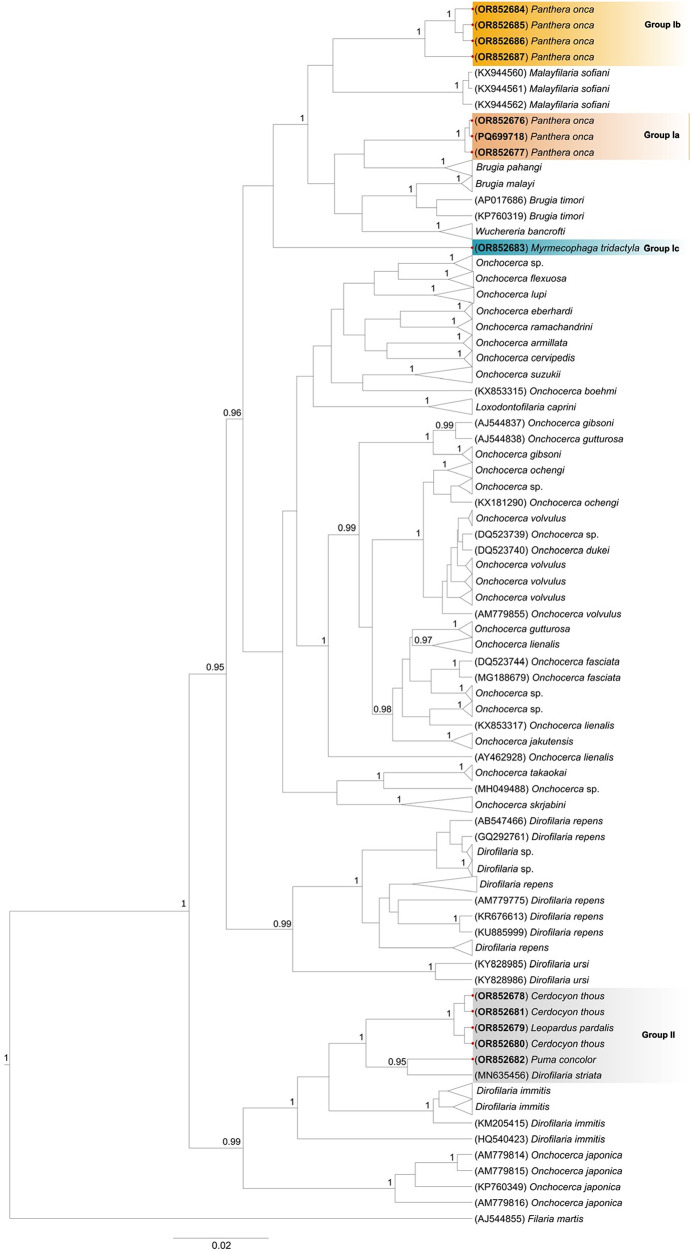
Bayesian phylogenetic inference of filarioid nematodes detected in different wild mammals based on the 12S rDNA gene. The outgroup used was *Filaria martis*. Complete list of accession numbers found in Supplementary File 2.

Based on the 12S rDNA gene sequences of the Onchocercidae family, the phylogenetic analyses grouped our sequences into 4 distinct lineages (Figure 2, Figure S1). Group Ia formed a sister clade to *B. pahangi*, while Group 1b formed a sister clade to *Malayfilaria sofiani*. The anteater sequence (Group Ic), while closely related to those in Group I, was more divergent and positioned as a sister group to this larger clade containing species within different genera: *Malayfilaria* (*M. sofiani*), *Wuchereria* (*W. bancrofti*) and *Brugia* (*B. timori, B. malayi* and *B. pahangi*).

All sequences from Group II clustered within the clade of *D. immitis* and *D. striata* (Figure 2). This group includes sequences from crab-eating foxes, an ocelot, and a puma. Also in Group II, 1 sample of puma (OR852682 #59) formed a sister clade with *D. striata*, represented by a sequence generated from a specimen isolated from a domestic cat from Florida, USA (MN635456). This puma *Dirofilaria* isolate appeared separate from the other puma sequences, which belong to Groups Ia and Ib.

Pairwise ML distances reinforced the patterns observed in the phylogenetic trees (Table 3).

**Table 3.** Pairwise maximum likelihood distances among 12S sequences of the family Onchocercidae, detected in free-living and captive wild animals in Brazil

Lineages	Average ML distances (range)
Among genera	
*Brugia* × *Malayfilaria*	0·0843 (0·0737–0·0958)
*Brugia* × *Wuchereria*	0·0964 (0·0520–0·1264)
*Malayfilaria* × *Wuchereria*	0·0750 (0·0617–0·0889)
Group Ia	
Group Ia × *Brugia*	0·0973 (0·0699–0·1237)
Group Ia × *Malayfilaria*	0·0802 (0·0732–0·0858)
Group Ia × *Wuchereria*	0·1078 (0·0852–0·1400)
Group Ia × Group Ib	0·1306 (0·1029–0·1559)
Group Ia × Group Ic	0·1565 (0·1504–0·1604)
Intra-group Ia	0·0000
Group Ib	
Group Ib × *Brugia*	0·1270 (0·0933–0·1768)
Group Ib × *Malayfilaria*	0·0949 (0·0800–0·1110)
Group Ib × *Wuchereria*	0·1418 (0·1025–0·2517)
Group Ib × Group Ic	0·1070 (0·0967–0·1154)
Intra-group IIb	0·0121 (0·0000–0·0256)
Group Ic	
Group Ic × *Brugia*	0·1400 (0·1083–0·1679)
Group Ic × *Malayfilaria*	0·0987 (0·0987–0·0987)
Group Ic × *Wuchereria*	0·1025 (0·0818–0·1342)
Group II (excluding Puma OR852682 #59)	
Group II × *Dirofilaria immitis*	0·0818 (0·0595–0·2060)
Group II × *Dirofilaria striata*	0·0548 (0·0395–0·0934)
Intra-group II	0·0027 (0·0000–0·0033)
Panthera OR852682 #59	
Panthera OR852682 #59 × Group II	0·0568 (0·0532–0·0621)
Panthera OR852682 #59 × *D. immitis*	0·0923 (0·0841–0·1034)
Panthera OR852682 #59 × *D. striata*	0·0375

## Discussion

Filarioid nematodes belonging to different genera were detected in samples from 5 out of 6 mammalian species. Based on the data obtained in this study, the presence of *Dirofilaria* species with zoonotic potential was recorded in wild cats. Using the mitochondrial 12S rRNA gene as a molecular marker, DNA of *Dirofilaria* was detected in both an ocelot and a puma. However, the phylogenetic position of the sequence obtained from the ocelot could not be clearly determined using only the 12S marker. Despite this limitation, the scientific literature indicates that parasites of the genus *Dirofilaria* infect a wide range of hosts and have previously been reported in different mammal species in the Americas. The described hosts include the jaguarundi (*Herpailurus yagouaroundi*), the jaguar (*P. onca*), several species of sloths (*Choloepus hoffmanni, Bradypus variegatus* and *B. tridactylus*) and the maned wolf (*C. brachyurus*) (Diaz-Ungria, 1973; Eberhard 1978a, 1978b; Trotti et al. 1997; Noronha et al. 2002; Deem et al. 2012; Fagundes-Moreira et al. 2024). In contrast, the sequence obtained from the puma clustered in a sister clade to *D. striata*. Notably, a similar observation was recently reported in Texas, USA, where the use of an alternative molecular marker (COI) led to the identification of *D. striata* in a specimen of bobcat (*Lynx rufus*) (Ramos et al. 2024).

Interestingly, when analysing filarioid sequences that form Group Ia (Figure 2), we observed that 3 jaguar sequences were allocated into a single group, closely related to the *B. pahangi*. Nevertheless, these may represent a previously uncharacterized species of *Brugia*. The current knowledge on the biodiversity within the genus *Brugia* is rather scarce, in particular those distributed across the Americas and associated with wildlife (Moraes et al. 2017; Kulpa et al. 2023). A thorough molecular characterization of *Brugia* isolates from different hosts and geographic locations is warranted for a better understanding of *Brugia* diversity and phylogenetic relationships among species. The occurrence of *Brugia* species in the Americas highlights their broad geographical distribution and the diversity of hosts for these filarioid nematodes. In North America, *Brugia* sp. has been reported in a dog (*Canis lupus familiaris*) in Canada (Kulpa et al. 2023). In the USA, *Brugia beaveri* is the most prevalent species, with infection records in raccoons (*Procyon lotor*), bobcats and possibly minks (*Neogale vison*). This wide variety of hosts suggests that *B. beaveri* has a remarkable ability to adapt to different ecological niches (Ash and Little, 1964; Beaver and Wong, 1988). Additionally, the identification of *Brugia lepori* in hares reinforces the hypothesis that lagomorphs play a significant role in the life cycle of these parasites in the region (Schlesinger et al. 1977; Eberhard et al. 1991). In South America, the identification of *Brugia guyanensis* in ring-tailed coatis (*Nasua nasua vittata*) from Guyana (Orihel 1964), as well as the detection of *Brugia* sp. in ring-tailed coatis from Brazil (Moraes et al. 2017), indicates that the region harbours a still underexplored diversity of filarioids.

When analysing sequences forming Group Ib (Figure 2), based on the 12S rDNA gene, the clustering of 4 Onchocercidae sequences from jaguars was observed, positioned as a sister group to the *M. sofiani*, a parasite of a rodent, the treeshrew (*Tupaia glis*) from Malaysia, closely related to *Brugia* and *Wuchereria* (Uni et al. 2017). A recent study by Fagundes-Moreira et al. (2024) also identified sequences of a filarioid nematode closely related to *Malayfilaria* in 2 jaguars (OR434083 and OR434084) in Brazil. This study characterized the COI gene, instead of the 12S gene targeted by us. A direct comparison was hindered as the cPCR targeting the COI gene for Group Ib samples was not successful. In light of this, the question arose as to whether these sequences represent the same species, as the samples were collected in the same region, the southern Pantanal of Mato Grosso do Sul.

Similarly, the identification of Group Ic, composed of the filarioid sequence (ID = 95·44% – OR852683 #9), was detected in a giant anteater from the municipality of Rio Negro, Mato Grosso do Sul, Brazil. However, the identification remained inconclusive, as in the phylogenetic analysis, the sequence was positioned separately from the larger clade containing the other sequences from Groups Ia and Ib. The ML distances between Group Ic and its closest lineages suggest a probable association with another genus. Therefore, the assignment of Group Ic to a specific genus also remains inconclusive.

Within the sequences obtained from puma, one (OR852682 #59) is substantially distinct from the others (Groups Ia and Ib), positioned as a sister group of *D. striata*. This pattern was confirmed by sequencing the COI region, which revealed a 6·3% divergence between the sequence of sample OR852682 #59 and that of *D. striata* found in a domestic cat from Florida, USA. This level of variation is notably high for intraspecific COI differences in *Dirofilaria* and other filarioid genera (Lefoulon et al. 2017; Kulpa et al. 2025). For instance, based on the available COI sequences in GenBank, the intraspecific divergence in *D. immitis* specimens does not exceed 1·5%, and in *D. repens,* it is limited to 1·1%. Hence, it is probable that the puma OR852682 #59 sample belongs to another *Dirofilaria* species closely related to *D. striata*, which is not yet represented in GenBank or could potentially represent a novel species.

Finally, Group II comprises samples from different hosts, including a canid (*C. thous*) and a felid (*L. pardalis*). This group comprises closely related and likely conspecific sequences, forming a sister clade to *D. striata*/ OR852682 #59 sequences. The average ML distance between Group II and *D. striata* was 0·0548 (0·0395–0·0934), while the average distance between Group II and puma 59 was 0·0568 (0·0532–0·0621). Therefore, these results suggest that the samples of *Cerdocyon* and *Leopardus* share the same *Dirofilaria* species, which is different from the one that infected the puma (Accession number: OR852682 #59).

Throughout the Americas, several species of the genus *Dirofilaria* have been reported from carnivore hosts through morphological, serological and molecular analyses. The canine heartworm, *D. immitis* has been found in a variety of wild carnivores, including gray foxes (*Urocyon cinereoargenteus*) (Simmons et al. 1980; Carlson and Nielsen 1983; Hernández-Camacho et al. 2016), raccoons (*Procyon lotor*) (Snyder et al. 1989; Ramos et al. 2024), black bears (*Ursus americanus*) (Crum et al. 1978), ring-tailed coatis (Moraes et al. 2017, 2022), oncilla (*Leopardus tigrinus*) (Filoni et al. 2009), coyotes (*Canis latrans*) (Zinck et al. 2021; Sobotyk et al. 2022), small-clawed Asian otters (*Aonyx cinereus*) (Upton et al. 2022) and wild felids (*P. onca, L. pardalis* and *Leopardus guttulus*) (Fagundes-Moreira et al. 2024). *Dirofilaria cancrivori* has been reported in crab-eating raccoon (*Procyon cancrivorus*) (Eberhard 1978b), and *Dirofilaria incrassata* from the subcutaneous tissues of both ring-tailed and crab-eating raccoons (Vicente et al. 1997; Guimarães et al. 2023), but no molecular data are currently available for this species. Another *Dirofilaria* species has been reported from the subcutaneous tissue of coatis, but questionably diagnosed as *D. repens*, a zoonotic species normally found in the Old World (Noronha et al. 2002; Capelli et al. 2018). While (*N. nasua*) *Dirofilaria lutrae* has been reported in the North American river otter (*Lontra canadensis*) (Orihel, 1965; Swanepoel et al. 2018), *Dirofilaria spectans* was found parasitizing the lungs and blood vessels of tayras (*Eira barbara*) (Noronha et al. 2002), Neotropical otters (*Lontra longicaudis*) (Noronha et al. 2002) and giant otters (*Pteronura brasiliensis*) (Vicente et al. 1997). The felid-associated *D. striata* was observed parasitizing margay cats (*Leopardus wiedii*) and pumas (*Puma concolor*) (Lentz and Freitas 1937; Forrester et al. 1985; Lamm et al. 1997; Vicente et al. 1997), as well as bobcats (Orihel and Ash, 1964; Miller and Harkema, 1968; Ramos et al. 2024). Finally, *Dirofilaria tenuis* was detected in raccoons (*P. lotor*) in North America (Sauerman and Nayar, 1985; Isaza and Courtney, 1988; Telford and Forrester, 1991; Richardson et al. 1992; Pung et al. 1996; Hernández-Núñez et al. 2024), which has been recently molecularly characterized. The lack of molecular data in many of these studies presents a challenge, particularly in light of recent revisions of the taxonomic framework for filarioids (Ferri et al. 2009; Lefoulon et al. 2015). This limitation has contributed to a gap in such studies in the Americas, as well as a paucity of records in Brazilian wild animals.

The genus *Dirofilaria* can also infect non-carnivorous mammals, with records of the species *Dirofilaria acutiuscula* and *D. striata* associated with the family Tayassuidae (Molin 1858). Furthermore, it is relevant to highlight the role of the superorder Xenarthra in infections caused by filarioid nematodes. The literature has described the occurrence of *Dirofilaria freitasi, D. incrassata,* and *Dirofilaria macrodemos* in the 3-toed sloth (*Bradypus tridactylus*) (Sandground, 1938; Mendonça, 1948), as well as *Dirofilaria panamensis* in the two-toed sloth (*Choloepus hoffmanni*) (Sandground 1938; Eberhard 1978a; Trotti et al. 1997). Currently, there is no molecular data for any of these *Dirofilaria* species associated with xenarthran hosts. The diversity of hosts that a parasite can infect increases the risk of it reaching new hosts, including domestic animals and humans.

According to the IUCN Red List (www.iucnredlist.org), the jaguar and maned wolf are near threatened, the giant anteater is vulnerable, and the crab-eating fox and the ocelot are of least concern. Considering the free-living habits of the animals and the data from this study, it is likely that these animals serve as reservoirs of pathogens, given the lack of knowledge about the arthropod vectors responsible for transmitting filarioid nematode third-stage larvae to wild mammalian definitive hosts in different biomes across Brazil. Various biological and logistic factors impact studies on parasite biodiversity, including filarioid nematodes, of wildlife in Brazil, including the remoteness of field sites, opportunistic sampling, funding allocation and conservation status of multiple host species. Additionally, the scarcity of molecular data available for many of the known, valid species within the genus *Dirofilaria* further hinders species-level characterization of taxa found through molecular screening of archival blood and tissue samples, in the absence of adult specimens or microfilariae in fresh blood samples. Therefore, studies using integrative taxonomy are essential to confirm the taxonomic status of these parasites and describe potential new taxa.

## Conclusion

Jaguars, puma, giant anteaters, crab-eating foxes and ocelots may play an important role as reservoirs for uncharacterized filarioid nematode species belonging to the family Onchocercidae. Currently, the impact of those infections on host health and their potential to infect companion animals and humans remains unknown. Furthermore, the fact that jaguars are infected by 2 different species of filarioid nematode species, likely belonging to distinct genera, highlights the complexity and significance of these parasites in the local fauna. Given the cryptic biodiversity of filarioid nematodes associated with Neotropical mammals, further studies are needed to assess their host and vector associations, geographic distribution and to elucidate their life cycle and pathogenicity.

## Supporting information

Santana et al. supplementary material 1 Santana et al. supplementary material

Santana et al. supplementary material 2 Santana et al. supplementary material

## Data Availability

Data supporting the conclusions of this study are included in the article. Generated sequences were submitted to the GenBank database under accession numbers: OR852676-87, PQ699718 and PQ699178.

## Supplementary material

The supplementary material for this article can be found at https://doi.org/10.1017/S0031182025101042.
