# Supplementary material for: Molecular detection of filarioid nematodes (Nematoda: Onchocercidae) in wild mammals from different Brazilian biomes
Source: Parasitology. 2025 Oct 30;152(13):1387–97. doi: 10.1017/S0031182025101042 (PMC12917412; doi:10.1017/S0031182025101042)
Supplement: Santana et al. supplementary material 1 — Santana et al. supplementary material [file S0031182025101042sup001.docx]

**Supplementary File 2:** Complete list of accession numbers of sequences of filarioid nematodes available on GenBank that were included in Figure 2 and Figure S1.

***Brugia pahangi***: AP017680, AM779851, KP760318, AM779849, AM779850, AJ544842.

***Brigia malayi***: MN598549, KP760317, AJ544843.

***Wuchereria bancrofti***: AJ544844, NC016186, AP017705.

***Onchocerca*** **sp.**: MN598548, MN598547, MN598546, MN598545, MN598544, AB518879, AB518877.

***Onchocerca flexuosa***: JQ733522, NC016172, DQ523746, ON854628, JQ733523, AP017692, DQ523747, JQ733521, LT732682, LT732683, ON854627.

***Onchocerca lupi***: KX853320, KC763784, MW266120, KP283475, HQ207645, JN863696, OL964949, KP283474, NC056960, GU365879, KP347443, KC686704, KC763783, KC686703.

***Onchocerca eberhardi***: KP760346, AM779810.

***Onchocerca ramachandrini***: KC167341, KC167340, DQ523737.

***Onchocerca armillata***: KP760345, KX853314, DQ523736.

***Onchocerca cervipedis***: JX075208, KX853316, JX075207.

***Onchocerca suzukii***: KX853321, AM779813, AM779812, AM779811.

***Loxodontofilaria caprini***: AM779819, AM779821, AM779822, AM779820, AM779818, AM779817.

***Onchocerca gibsoni***: AY462912, AY462928.

***Onchocerca ochengi***: KC167333, KX181289, NC031891, AY462916, DQ523742, AP017694, KC167334, KC167330, KC167335, AP017693, KC167331, AY462918, AY462915, FM206484, KC167332, KP760348, AJ544839, AY462914, AY462917.

***Onchocerca* sp**.: DQ523738, KC167338, KC167337, KC167336.

***Onchocerca volvulus***: AJ544840, KC167339.

***Onchocerca volvulus***: AM779853, NC001861, AP017695, AM779852, DQ523741, AM779854, KT599912, AY462920, AY462921, AY462919.

***Onchocerca gutturosa***: AY462923, AY462922, DQ523743, KP760347.

***Onchocerca lienalis***: AY462926, AY462927, KX853318, AY462925, AY462924.

***Onchocerca* sp.**: JX075210, JX075211, JX075213, JX075215.

***Onchocerca jakutensis***: HQ717719, ON854631, DQ523745, ON854629, ON854630, HQ717720, ON854632.

***Onchocerca takaokai***: AB972363, AB972364, AB972362.

***Onchocerca skrjabini***: ON854635, ON854637, ON854634, ON854636, ON854633, AM779804, AM779807, AM779806, AM779808, AM779809, AM779805.

***Dirofilaria* sp.:** KX265050, MZ810545, NC031365, KX265092, KX265093.

***Dirofilaria repens***: KM205385, KX265047, KX265064, KX265053, KF494237, KM205387, KM205411, KC953031, KM205382, AM779777, KR071802, KX265070, KR780979, KM205410, KX265090, KX265048, KX265049, NC029975, KM205380, KX265073, KX265074, AB973228, KM205404, KX265063, KX265068, KM205386, KX265081, KX265067, KX265071, KM205406, KX265091, KX265052, KX265076, KM205408, KM205405, KX265072, JF461462, AM779772, KX265089, KP898737, AM779778, KX265075, KX265080, KM205377, KX265060, KM205402, KM205403, KX265059, KX265061, KX265078, KX265062, KM205409, KX265065, KX265082, KX265056, KM205388, KX265069, KM205395, KM205399, KP250992, KX265086, KM205384, KM205397, KX265087, KY828983, KM205396, KX265085, KM205389, KM205394, KM205373, KX265083, KM205378, KM205401, KM205374, KX265066, KM205381, KM205393, KM205398, KX265051, KX265058, KX265057, KX265079, KM205383, KX265088, KM205372, KM205375, KY828984, KP760331, KM205392, KM205407, KM205400, KM205391, KX265077, KM205379, KX265054, KM205376, KX265055, KM205390, KX265084, KP898736, KR676613, KU885999, AM779775, AM779773, AM779774, AM779776, AJ544832.

***Dirofilaria immitis***: FN391554, KF707482, MH051846, EU182328, AJ544831, KF707478, KF707480, KF707479, KP760330, AM779770, KF707476, JX502021, MF059093, EU169125, AM779771, KF553637, KF707477, KF707481, KM205412, KM205413, KM205414, OL714337, AM779769, KU885998, KP898738, EU182327, KM205145.
