# Supplementary figures and images for: Molecular detection of filarioid nematodes (Nematoda: Onchocercidae) in wild mammals from different Brazilian biomes
Source: Parasitology. 2025 Oct 30;152(13):1387–97. doi: 10.1017/S0031182025101042 (PMC12917412; doi:10.1017/S0031182025101042)

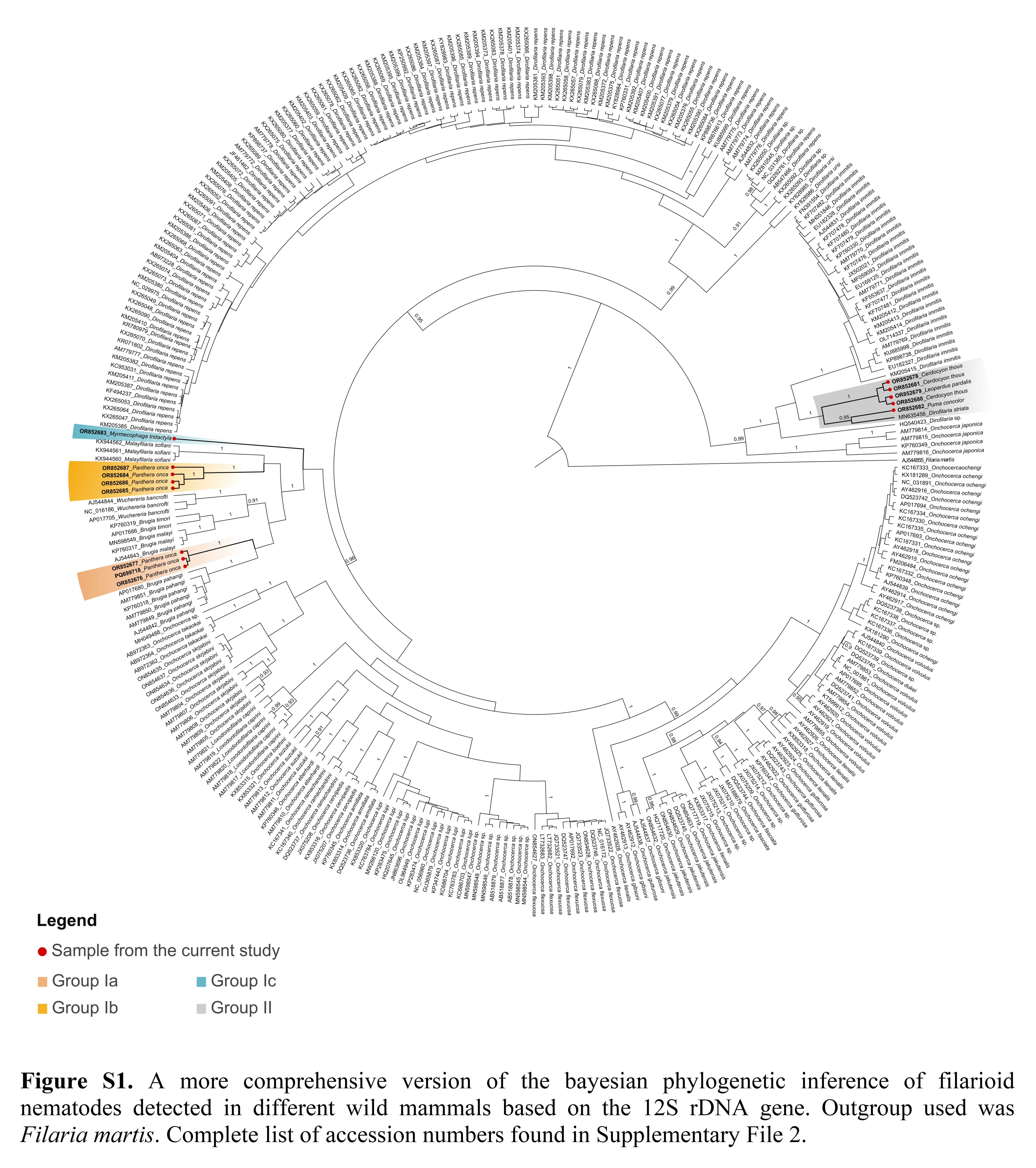

Supplement: Santana et al. supplementary material 2 — Santana et al. supplementary material [file S0031182025101042sup002.jpeg]
